# Flipped online teaching of histology and embryology with design thinking: design, practice and reflection

**DOI:** 10.1186/s12909-024-05373-7

**Published:** 2024-04-09

**Authors:** Yan Guo, Xiaomei Wang, Yang Gao, Haiyan Yin, Qun Ma, Ting Chen

**Affiliations:** 1 College of Basic Medicine, Jining Medical University, 133 Hehua Road, Jining, 272067 China; 2 Academic Affair Office, Jining Medical University, 133 Hehua Road, Jining, 272067 China

**Keywords:** Flexible hybrid teaching, Design thinking, Flipped teaching, Histology and embryology, Basic Medical Education

## Abstract

**Background:**

Flexible hybrid teaching has become the new normal of basic medical education in the postepidemic era. Identifying ways to improve the quality of curriculum teaching and achieve high-level talent training is a complex problem that urgently needs to be solved. Over the course of the past several semesters, the research team has integrated design thinking (DT) into undergraduate teaching to identify, redesign and solve complex problems in achieving curriculum teaching and professional talent training objectives.

**Methods:**

This study is an observational research. A total of 156 undergraduate stomatology students from Jining Medical University in 2021 were selected to participate in two rounds of online flipped teaching using the design thinking EDIPT (empathy, definition, idea, prototype, and test) method. This approach was applied specifically to the chapters on the respiratory system and female reproductive system. Data collection included student questionnaires, teacher-student interviews, and exam scores. GraphPad Prism software was used for data analysis, and the statistical method was conducted by multiple or unpaired t test.

**Results:**

According to the questionnaire results, the flipped classroom teaching design developed using design thinking methods received strong support from the majority of students, with nearly 80% of students providing feedback that they developed multiple abilities during the study process. The interview results indicated that teachers generally believed that using design thinking methods to understand students' real needs, define teaching problems, and devise instructional design solutions, along with testing and promptly adjusting the effectiveness through teaching practices, played a highly positive role in improving teaching and student learning outcomes. A comparison of exam scores showed a significant improvement in the exam scores of the class of 2021 stomatology students in the flipped teaching chapters compared to the class of 2020 stomatology students, and this difference was statistically significant. However, due to the limitation of the experimental chapter scope, there was no significant difference in the overall course grades.

**Conclusion:**

The study explores the application of design thinking in histology and embryology teaching, revealing its positive impact on innovative teaching strategies and students' learning experience in medical education. Online flipped teaching, developed through design thinking, proves to be an effective and flexible method that enhances student engagement and fosters autonomous learning abilities.

## Research background and motivation

Histology is the study of the microstructure and related functions of the human body [1], while embryology studies the laws and mechanisms of ontogenesis and development; these two sciences are interrelated and self-contained [2]. As one of the important professional core programs of most medical specialties, Histology and Embryology (HE) has been an indispensable curriculum bridge between normal microstructure and pathological changes in tissue and organs.

The teaching targets of HE are mainly first-year undergraduate students in clinical medicine, psychiatry, stomatology, nursing, etc. The importance of fostering the development of empathy in undergraduate students is continuously emphasized in international recommendations for medical education [3]. Freshmen have a certain ability to think logically and analyse problems, but this ability is limited, and they have a yet to develop familiarity with scientific research hotspots. Moreover, they are often unaware of their creative potential, and this phenomenon often causes them to passively accept knowledge, and their autonomous learning ability and student participation in class are less than that of upperclassmen. These first year students face the need to develop scientific literacy and the ability to integrate theory with practice [4]. However, traditional teaching methods may have failed to fully meet students' need for a profound understanding of these abstract concepts, leading to challenges such as low interest in learning and inadequate knowledge absorption. Consequently, educators urgently need to seek innovative teaching strategies to enhance students' learning experience and academic performance.

In the information age, teacher teaching is no longer a simple superposition of knowledge and teaching methods but a fusion innovation of technology and teaching oriented to a more complex learning environment. The Teacher Standards issued by the American Educational Technology International Association note that the important role of future teachers is that of a "designer" [5]. DT combines a creative and innovative approach to dealing with complex problems, which serves as a valuable tool for those seeking to improve the challenging issues in medical education [6]. DT is a process of analysis that relies on the deconstruction of ideas and a creative process that relies on the construction of ideas. There are no judgements in DT. This eliminates the fear of failure and encourages maximum input and participation. Wild ideas are welcome since they often lead to the most creative solutions. Everyone is a designer, and DT is a way to apply design methodologies to any situation [5].

In the field of education, DT has been advocated as a means to promote the cultivation of innovative talent through innovative teaching methods. With the help of DT, and adhering learning as the concept in teaching, the transformation of teaching allows learners to explore real needs in real life scenes, to propose innovative solutions to meet those needs through teamwork, and to test the effectiveness of those solutions through prototype production. This process facilitates the further application of constructivism [7].

**Fig. 1:**
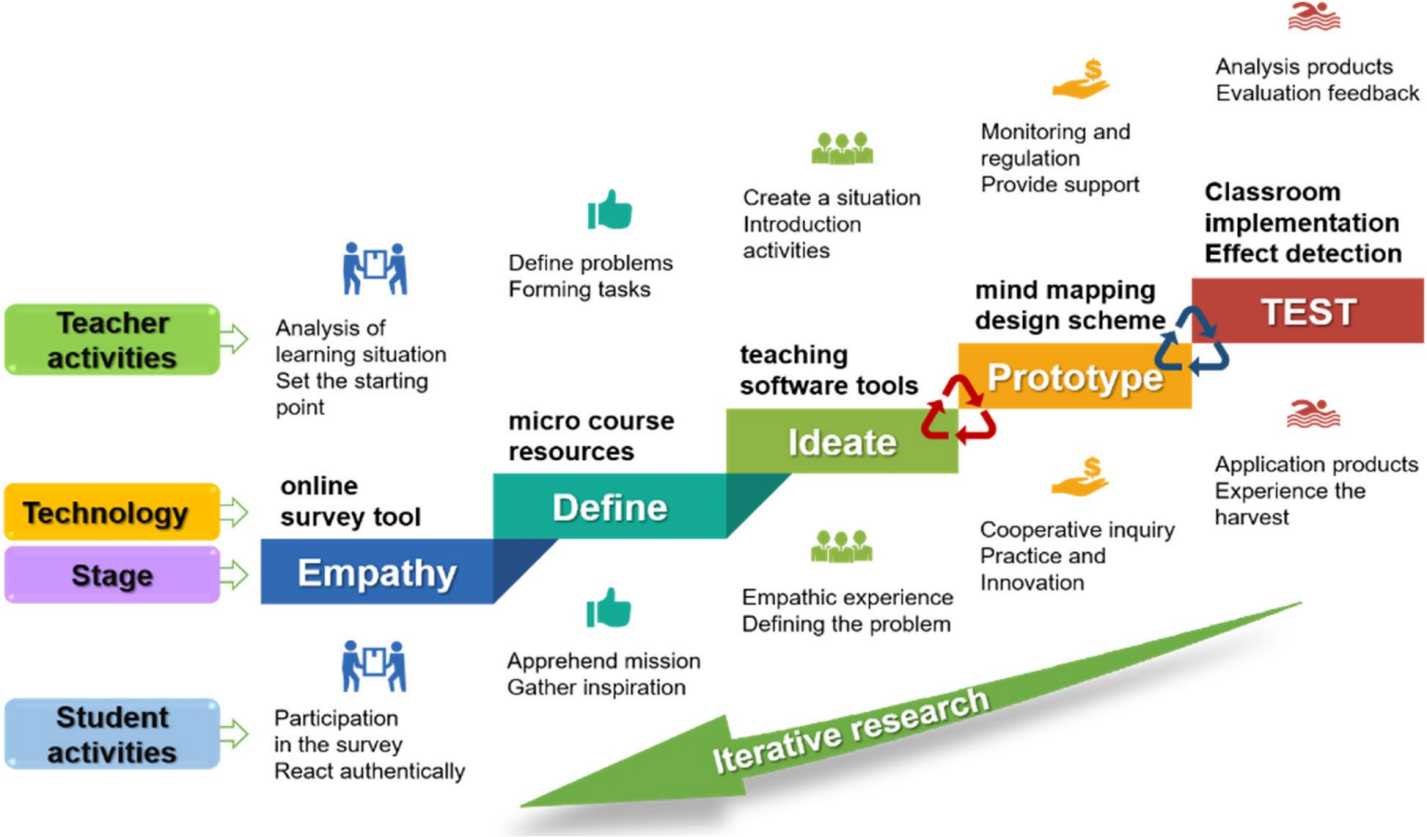
Problem solving steps incorporating DT

In the process of both conventional teaching and teaching innovation, the research team utilizes the “EDIPT” (Empathy, Define, Ideate, Prototype and TEST) DT theory [8] which originating in the Stanford University Design School to design teacher activities and student activities and select technical tools [9]. The basic process is shown in Fig. 1. The team is very accustomed to consciously applying DT methodology when facing difficulties and challenges to consistently obtain the desired results [10]. This study sets the teaching objectives and plans of a large cycle (one semester) to guide the teaching implementation of a small cycle (one section or one chapter); Then, small-cycle teaching feedback and achievement accumulation promote the progress of large-cycle teaching to ensure the coherence, effectiveness and improvement of teaching reform. For example, the difficult problem in the process of cardiovascular system embryogenesis is atrial separation; the team uses cardboard and plastic film to construct room partition "products" [11] to provide vivid explanations and body movements for clearer explication. In another example, they integrate scattered knowledge points including cleavage, blastocyst formation and implantation into a unified narrative called "the initial journey." It solves the pain point that the dynamic abstraction of embryology knowledge is difficult to intuitively understand. The above are two examples of using EDIPT steps of design thinking to solve teaching pain points.

### Research objectives and significance

In the 2021 Horizon Report: Teaching and Learning Edition, blended learning was once again selected as the key technology affecting the future development and practice of higher education [12], demonstrating great application potential. In this format, the teaching team adheres to the following practical principles to promote more blended learning courses to ensure high-quality outcomes [13]. In the recent period of epidemic prevention and control, effective online teaching combines asynchronous and synchronous delivery modes, addresses knowledge learning and ability development, and highlights interaction in teaching activities to improve the online teaching experience for both teachers and students and enhance the overall quality of online teaching. Online teaching is not simply an emergency measure taken during the epidemic but rather represents the future trend of education.

The aim of this study is to explore the application of design thinking in the teaching of histology and embryology courses. By investigating the impact of design thinking in the teaching process, we aim to gain a deeper understanding of the effects of this innovative teaching strategy on students' learning experience and academic performance, as well as its potential applications in medical education.

The significance of this research lies in its contribution to medical education with novel teaching methods and strategies. By incorporating design thinking, educators can better cater to students' learning needs and enhance their comprehension and mastery of the subject matter. Furthermore, this study contributes to the expansion of teaching research in the field of medical education, providing valuable insights for educational reform and improvements in teaching quality.

### The analysis of the correlation between design thinking and this study

Design thinking plays a crucial role in formulating the educational reform. During the empathize phase, an in-depth understanding of teachers' and students' needs and challenges is achieved. This includes considering teachers' expectations and pedagogical beliefs, as well as students' learning styles and feedback, leading to a clear definition of the problem and setting specific objectives for the educational reform. In the define phase, the importance of improving teachers' pedagogical approaches and methods, and cultivating students' creative learning and competencies is underscored. This serves as the foundation for selecting appropriate teaching strategies and establishes the specific direction for incorporating design thinking in the flipped classroom model. During the ideate phase, innovative thinking is employed to explore diverse teaching strategies. For enhancing teachers' pedagogical approaches, approaches such as case-based teaching and collaborative learning are recommended to stimulate students' intrinsic motivation for active learning. For promoting students' creative learning and overall competencies, methods like project-based learning and critical thinking cultivation are considered to facilitate holistic student development. In the prototype phase, the devised teaching strategies are implemented in the flipped classroom setting. Continuous prototyping and rapid experimentation facilitate the collection of valuable feedback and data from students and teachers, enabling further optimization of the teaching strategies to align with the original intent of design thinking. Finally, in the test phase, a comprehensive evaluation of the teaching implementation is conducted. By collecting and analyzing data, the study delves deep into the impact of the educational reform on teachers' pedagogical beliefs and students' creative learning and overall competencies. This process provides crucial feedback and evidence for the ongoing improvement of the educational reform.

In conclusion, the selection of flipped classroom as a pedagogical strategy is closely guided by design thinking principles. Through the application of design thinking, this observational study aims to enhance teachers' pedagogical approaches and methods while fostering students' creative learning and overall competencies, thus promoting the successful implementation of the educational reform.”

Flipped classroom sessions can also allow learners to gain competence through their educational endeavours [14]. As Bransford writes, “To develop competence in an area, students must: a) have a deep foundation of factual knowledge, b) understand facts and ideas in the context of a conceptual framework, and c) organize knowledge in ways that facilitate retrieval and application” [15]. Flipped classrooms can lead to competence in factual knowledge by fostering mastery of content through content understanding and application, as in traditional classrooms [16].

### “O-PIRAS” Flipped classroom

The flipped classroom teaching model used in this study was formed and adjusted on the basis of Professor Jianpeng Guo's “O-PIRTAS” model. The flipped teaching mode can enable both teachers and students to acquire further abilities through teaching activities [17].

**Fig. 2:**
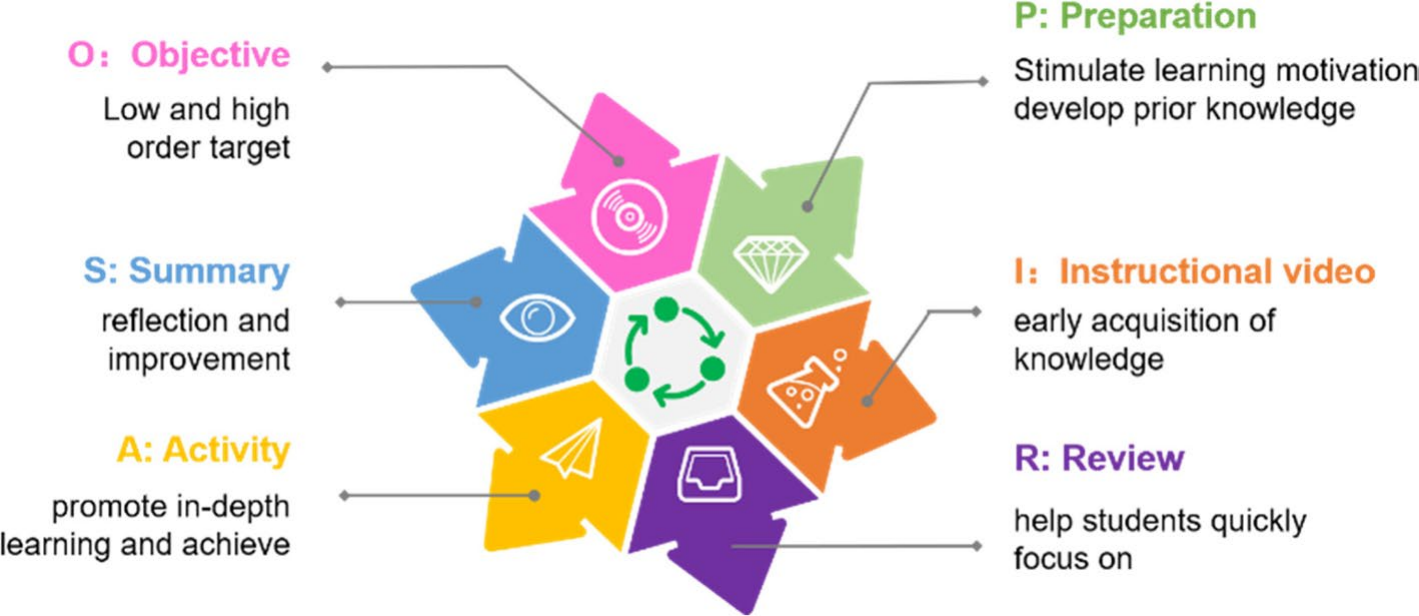
Process of “O-PIRAS” flipped teaching

The first step(O: Objective) in flipped classroom teaching design is to formulate two types of teaching objectives: low level and high level. The lower level teaching objectives include two cognitive objectives from Bloom's classification of teaching objectives: the memory and understanding of knowledge, while the higher level teaching objectives include four cognitive objectives from Bloom's classification: application, analysis, evaluation and creation, as well as objectives pertaining to movement skills and emotion [18]. The second step is to design a preparation activity (P: Preparation) for students to complete before class, which helps students form necessary prior knowledge and stimulates their learning motivation by exploring relevant issues prior to the class [19]. The third step is for teachers to send teaching materials (I: Instructional video) to their students for pre-class learning to facilitate their early acquisition of knowledge [19]. Fourth, teaching is transferred from online classes to offline classes. The teacher briefly reviews (R: Review) the video content before class to help students quickly focus on and prepare for the next stage of learning both cognitively and psychologically. Fifth, teachers should design classroom activities (A: Activity) appropriate to high-level teaching objectives to promote in-depth learning and successfully achieve high-level objectives. Sixth, teachers should conduct classroom summaries (S: Summary), reflection and improvement to help students form integrated structured knowledge. The six steps of flipping the classroom link form a closed loop, which can be summarized as in Fig. 2.

## Methods

### Research method and data collection

Conveniently selecting 156 undergraduate students majoring in Dentistry from the 2021 cohort of Jining Medical University, we designated classes 1 to 3 as the class of 2021 stomatology students. As the class of 2020 stomatology students, we chose 155 undergraduate students majoring in Dentistry from the 2020 cohort, also from classes 1 to 3. Prior to the start of the study, we conducted communication sessions with both teachers and students, ensuring that all students were well-informed about the study and provided their consent. The two groups of students had the same course hours, faculty resources, learning materials, and learning spaces. The only difference was the application of design thinking methods in course and teaching design, including the implementation of flipped classroom teaching, specifically tailored for the 2021 cohort of students.

Data collection was conducted through various methods, including distributing questionnaires, conducting pre-, mid-, and post-research interviews, and recording course and corresponding chapter test scores. The implementation chapter selected the respiratory system, which plays a bridging role within histology, and the female reproductive system, which plays a transitional role between histology and embryology.

Before studying "Respiratory System", students have already mastered the basic methods of using design thinking to learn histology, and have a deep understanding of the four basic tissues and two types of organs (hollow and substantial organs). The main organs of the respiratory system—the trachea and lungs—belong to two types, respectively. The female reproductive system, as the concluding chapter of histology, is separated from the flipped classroom of the respiratory system by two weeks, leaving appropriate time for teachers to iteratively design and students to adapt to new methods. Four surveys were administered during the research process: Pre-flipped classroom survey for Chapter 16 "Respiratory System", Post-flipped classroom survey for Chapter 16 "Respiratory System", Pre-flipped classroom survey for Chapter 19 "Female Reproductive System", and Post-flipped classroom survey for Chapter 19 "Female Reproductive System", to gather student feedback and opinions on the teaching methods. The questionnaires were designed based on the research objectives and questions, and were refined through pre-testing to ensure clarity, accuracy, and appropriateness of the questions and options. The questionnaire mainly includes the following dimensions: ⑴Basic information of students, Q 1–3; ⑵ Learning and satisfaction: Q 4, What is the division of labor in your group in this cooperation? Q 7, About flipping class, how long will you spend studying before class? Q 6 Compared with the last flip class, are you satisfied with the teacher's teaching time in this flip class? Q 12, What are you most satisfied with this flip class? (3) Learning experience and ability improvement: Q 5, What kind of class learning form do you like best in flip class? Q 8, What are your learning pain points or difficulties after this flip class? Q 9, What abilities have you improved in this flip class? ⑷ Classroom Improvement and Feedback: Q 11, What are the advantages of this flip class compared with the last flip class? Q 10, In the course of embryo formation, do you like to use flip class for multiple course contents? Q13, What suggestions do you have for improving the embryo flipping class? Interviews were conducted at various stages, including before the study to understand teaching pain points, during the research process to gauge teachers' and students' attitudes and perspectives on the teaching activities, and after the study to obtain overall feedback. Additionally, we conducted both stage-specific and overall tests, and promptly collected relevant data for comparative analysis with the class of 2020 stomatology students. These data provided comprehensive insights into the performance and experiences of students in both the experimental and class of 2020 stomatology studentss.

## Application of design thinking in course design

In course design, we employed design thinking methods to redesign the histology and embryology curriculum. Firstly, we gained a deep understanding of students' learning needs and interests to define course objectives and content. Secondly, we innovatively designed online materials and videos to enhance the appeal and practicality of the learning experience. We encouraged students to actively participate in discussions and problem-solving during class to unleash their creative potential. Additionally, we continuously optimized the teaching content and methods through iteration and feedback to ensure a sustained improvement in teaching effectiveness. Through the application of design thinking in course design, we expected to optimize the teaching process, enhance students' learning experiences, and improve their academic performance.

### Design and implementation of flipped teaching

The HE course covers 22 chapters, totaling 60 h, including 44 h of theoretical classes and 16 h of practical classes. The theoretical teaching is roughly divided into three stages: the first stage consists of 12 h, focusing on introducing the four basic human tissues; the second stage comprises 18 h, covering the structure of human organs and systems; and the third stage spans 14 h, elucidating the process of human embryonic development. To facilitate a deep understanding and mastery of human tissue structures, four practical classes, each lasting 4 h, are incorporated to complement the theoretical content.

The entire course relies on a blended teaching approach, combining online and offline instruction, leveraging the resources of Shandong's top undergraduate course in HE, and utilizing the "Zhidao" flipped classroom tool. At the beginning of the course, the teachers introduce the purpose, teaching process, weekly plan, grading components, and assessment methods of incorporating design thinking into the blended HE teaching. The flipped classroom teaching for the class of 2021 stomatology students is set between two stage tests to investigate whether this innovative teaching method has an impact on students' test scores.

The teaching team consists of 4 associate professors and 3 lecturers, with an average teaching experience of 11.4 years in teaching nursing major foundation courses and possessing rich teaching experience. In addition, the project leader and team teachers have undergone multiple training sessions in design thinking innovation and systematic training in domestic and on-campus blended teaching theories.

At the beginning of the semester, the curriculum teaching plan should be formulated, and chapters suitable for flipped teaching should be selected according to the teaching plan” and content characteristics [20]. Teaching and research team members should jointly analyse the teaching content and formulate the flipped classroom syllabus [21], clarify teaching objectives (knowledge objectives, ability objectives and emotional objectives, i.e., low-order objectives and high-order objectives), develop chapter teaching plans and teaching courseware (traditional classrooms are obviously different from flipped classrooms) [22], record pre-class video (design the course content in a fragmented way and systematically present it in accordance with the teaching plan) [23], divide students into groups and engage with all students through “zhidao” teaching software and the QQ class committee. The specific design and implementation plan for the preparation of the above teaching materials for a flipped classroom course on the respiratory system. The teaching team seminar is held three weeks before the class.

While completing the preparation of teaching materials in accordance with the teaching plan, the team clarified what methods and tasks teachers and students should complete before and during the implementation of the flipped classroom so that everyone can understand the design intent of these teaching activities to facilitate more satisfactory teaching results.

### Practice processes and instructional evaluation

The teaching design was discussed and approved by all members of the research team and used in the classroom teaching of respiratory system conversion with slightly modified specific content. One week before class, it was distributed through the zhizhuishu teaching platform to all the students [24] participating in flipped classroom teaching. The resources provided to students include preview materials, textbook chapters, courseware, videos, etc.; Preview questions, some questions related to preview materials, guide students to think and explore, stimulate learning interest and initiative; Learning objectives, clarify the knowledge objectives, ability objectives, and literacy objectives for pre class learning. In addition, there are also learning platforms (Wisdom Tree Online Course-https://coursehome.zhihuishu.com/courseHome/1000007885/199185/20#onlineCourse),WeChat class group chat, learning community. In flipping the method of respiratory system class delivery, the team first tried to perform a complete flip of the class. At the beginning of the class, the teacher clarified six themes, and then the group spokespersons demonstrated their understanding of all the knowledge points, including key points and difficulties, in class by drawing lots. The teams provided feedback for each other. The teacher only played a guiding role in the activities involving the entirety of the class. After summarizing the classroom content, the teacher assigned homework, such as creating mind maps and engaging in thematic discussions on the learning platform, and distributed the questionnaire regarding the group pre-class preparations, classroom activities and learning experiences for the respiratory system flipped classroom. The questionnaire mainly consists of the following questions. How was the work divided among your team for this activity? What kind of in-class learning style do you like best in the flipped classroom? Compared with the last flipped classroom, are you satisfied with the length of teaching in this flipped classroom? How long do you spend on pre-class learning for a flipped class? What are your learning pain points or difficulties after this flipped lesson? What abilities have you improved in this flipped classroom? Are you satisfied with the length of lectures in this class compared with that in the last flipped class? What percentage of the course content do you prefer to be delivered by the flipped classroom model? Compared with the last flipped classroom, what are the advantages of this flipped classroom? What you are most satisfied with in this flipped lesson? Please offer suggestions for the improvement of your flipped class on embryos.

According to the steps and links involved in DT, when the “product” (teaching plan) is tested and problems are found, the design team should complete the iteration as soon as possible to better meet the needs of “customers” (students)[25, 26]. Three days after the questionnaires, the teaching team adjusted the flipped classroom teaching design scheme for the Female Reproductive System course according to the questionnaire results, and arranged the pre-class tasks one week prior to the class, which differed from the previous class. Explanations of key points and difficult points were appropriately added to the teaching design, which did not depend on students as thoroughly as it had the last time, reducing the difficulty of the flipped classroom to a certain extent, improving students' level in participation, and improving the learning effect and teaching quality of the class.

A total of four questionnaires were distributed before and after the two flipped classes, and video recordings were made of the flipped classroom teaching process for a nursing and a stomatology class. Tencent conference recording instructions were issued by teachers. HE course scores consisted of three parts, including the usual score (30%), experimental score (10%) and final score (60%). The course scores of the 2021 nursing class and stomatology class were derived from the education management system of Jining Medical College, and the course scores of the nursing and stomatology majors who did not classes that had implemented online flipped classroom teaching in 2020 were derived as a control. Comparing the proportion of students in each of two grades, the total correct response rate of test questions, and the correct response rate of respiratory system and female reproductive system course test questions delivered through flipped classroom teaching were analysed using GraphPad Prism software through the statistical method of multiple or unpaired t tests.

## Results

### Teaching strategies developed using design thinking methods improves multiple student abilities

According to the results of the questionnaire distributed before the beginning of the first flipped class, 51.2% of the students reported not understanding the new learning method and that they could not check the data, 21.6% of the students were not interested in flipped lessons and preferred traditional passive learning methods, 25.6% of the students said that they did not have strong self-control and were unwilling to take the initiative to learn, 56.8% of the students said that they had a great fear of speaking in front of their classmates and that their public speaking skills were not strong, and 46.4% of the students did not know how make suitable PowerPoint Presentation (PPT). After two sessions of flipped classroom learning, the majority of students felt that their pain points had been effectively solved and various abilities had been developed. The results of the question after the flipped classroom teaching of the female reproductive system are shown in Table 1.

**Table 1:**
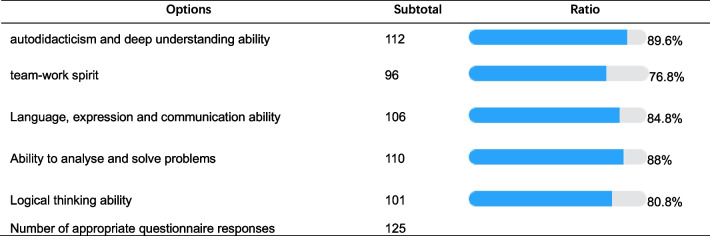
Results of the Question “What abilities have you improved in this flipped classroom?”

### Positive feedback and growth experience of students in teaching strategies developed using design thinking methods

The informal discussion following the flipped lesson on the female reproductive system shows that compared with the "Teacher almost let go" response in the previous respiratory system flipping class, the students are more inclined to respond with "The teacher will solve the problems left in our preview," "Feedback is provided between groups, and the groups are complementary," "The teacher emphasizes the key points, explains the process in detail, and plays videos to consolidate knowledge," and " the teacher commented on the performance of the group speaker". The students thought that after two sessions of participation in a flipped classroom, "We are more active in learning and the classroom design is more live," and "The students are more involved and confident." "By applying design thinking to study the course of organizational design, I have found new learning methods and approaches, and successfully applied these learning methods to other courses, which has benefited me greatly."

### The comparison results of grades

**Fig. 3:**
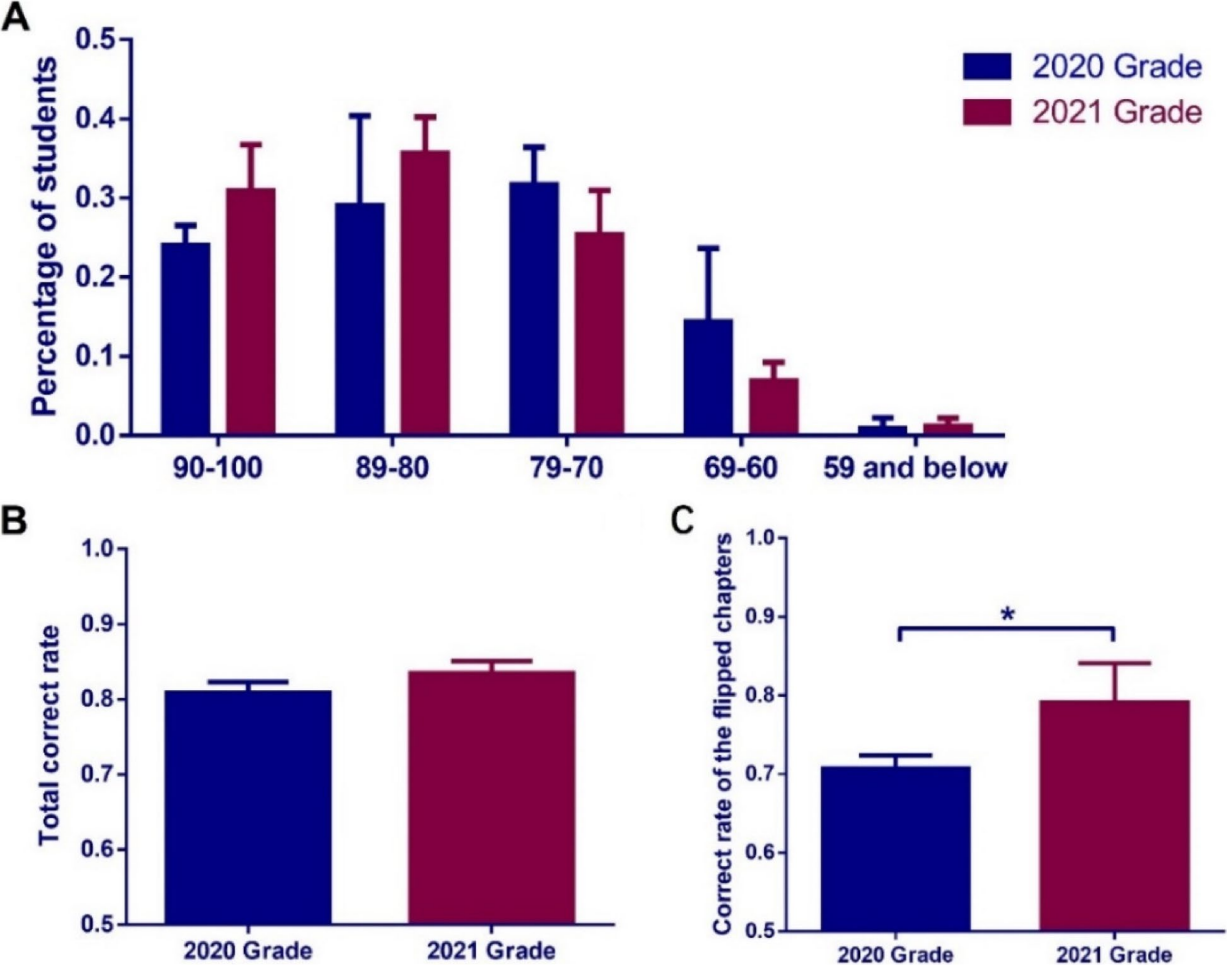
Distribution of final exam scores for the two graduating classes. **A** The proportion of students in different grades, no significant difference Statistical method: Multiple t tests. **B** Total accuracy, no significant difference. Statistical method: Unpaired test. **C** The accuracy of flipped classroom chapters, unpaired test, *P* < 0.05. Mean ± SEM of column A 0.7075 ± 0.009587 *N* = 3, Mean ± SEM of column B 0.7913 ± 0.02872 *N* = 3

Under the premise that there is no significant difference in the difficulty of test questions and other criteria between the flipped and traditional classrooms, the class of 2021 stomatology students' course scores showed a slight improvement. However, there was no significant difference in the distribution of the number of students in each score segment compared to the class of 2020 stomatology students. In contrast, for the chapters that implemented flipped classroom teaching, specifically the respiratory system and female reproductive system chapters, the class of 2021 stomatology students' test scores showed a significant increase. The difference between the two groups was statistically significant. The details are depicted in Fig. 3.

## Discussion

Firstly, significant achievements have been made in enhancing students' overall abilities through the application of design thinking methods in formulating flipped classroom teaching strategies. Preliminary surveys revealed various challenges faced by students before the commencement of the flipped classes, including difficulties in understanding new learning methods, lack of interest in flipped classes, low self-discipline, and fear of public speaking. However, after two sessions of flipped classroom learning, the majority of students believe that their pain points have been effectively addressed, and various skills have been developed. This aligns with the findings of previous research by Awan OA [15], indicating that the application of design thinking methods in teaching strategies can significantly enhance students' subject engagement and skill development.

Secondly, regarding the positive feedback and students' growth experiences in applying design thinking methods to formulate teaching strategies, there is a positive trend observed in informal discussions following the flipped classroom on the female reproductive system. Students tend to perceive a more proactive role played by teachers in the flipped classroom, addressing the issues they encountered during previewing. Students also highlighted the complementary feedback provided among groups, emphasizing the importance of teamwork. Additionally, students positively acknowledged the efforts of teachers in emphasizing key points, providing detailed explanations of processes, and reinforcing knowledge through video presentations. They believe that this teaching approach stimulates their interest in learning and enhances their motivation. This aligns with the findings of research by Scheer A [7] and Deitte LA [11], supporting the positive impact of design thinking methods in education.

Finally, the results of the performance comparison indicate that there is no significant difference between flipped classroom and traditional classroom based on criteria such as question difficulty. However, the overall grades of the 2021 cohort of dental medicine students have shown a slight improvement. Specifically, in the chapters on the respiratory and female reproductive systems within the flipped courses, the exam scores of the 2021 cohort students have significantly increased, and this difference is statistically significant. This suggests that the flipped classroom teaching formulated through design thinking methods has a significant positive impact on the development of subject-specific skills in specific chapters. This aligns with the relevant findings of Cheng X [1], further emphasizing the instructional advantages of design thinking methods in specific topics.

### Main finding

The team used DT to reveal the pain point that flexible mixed teaching can not guarantee students' participation and the realization of teaching objectives, and the application of online flip classroom teaching solved this problem well Students play a leading role in this kind of teaching, so they need to devote more time and energy to preview textbooks and consult relevant materials before class to improve their autonomous learning ability It is helpful to cultivate team spirit in flip teaching in the form of group, which is helpful to cultivate team leadership and management ability. The main requirements of mixed teaching are to integrate pre-recorded videos into the course as a whole and provide online learning resources to supplement face-to-face teaching in an organized and selective way [27] As assessment expert Mag says, if you are teaching something that cannot be assessed, you are already in an awkward position-that is, you can't explain the teaching content clearly [28] Therefore, reasonable teaching objectives in mixed teaching can make teachers and students reach a common understanding and consensus on learning results, enhance emotional communication and resonance between teachers and students, and jointly promote the implementation of curriculum teaching. The successful implementation of online flip class needs certain network and students' enthusiasm and cooperation At the same time, teachers need to be particularly familiar with the curriculum to design lectures and targeted comments [29].

### Limitations and future research

In this study, the respiratory system and female reproductive system in HE were selected as subjects for conducting flipped classroom teaching. The examination results shoe that although the overall course performance has not significantly improved, the accuracy of the chapter test questions in flipped classrooms significantly improved, which demonstrates that this teaching method can improve students' learning performance while cultivating their various abilities. It is worth expanding the scope of implementation to more chapters. However, not all chapters are suitable for flipped classroom teaching. Because the two chapters involved in this paper belong to the "organs and systems" module, it does not fully reflect the applicability of this research in this course. Some chapters of the basic tissue module and embryogenesis module are also the scope of our future teaching research In addition, what is the highest proportion of total course hours converted to flip teaching? All these problems need further study in the future. What is the most appropriate ratio of total course hours to convert into flipped teaching? These issues need to be further studied in the future.

When DT is introduced into education, evaluating students' learning and development becomes more important than evaluating students' design products or knowledge and ability. Changes in consciousness and attitude include whether they can fully participate in current cognitive activities, learn independently, communicate and cooperate, and continuously monitor and adjust themselves. By clarifying this guidance, we can formulate or select appropriate evaluation criteria through a literature review during the implementation of the project and adjust the subsequent research conditions in a timely manner according to the evaluation results.

Online flipped teaching is an effective way to integrate DT into the flexible and mixed teaching of HE, which can effectively enhance students' learning input and cultivate students' autonomous learning ability. This research aims to reshape the method of classroom teaching through the deep integration of modern information technology into pedagogical design. Future work should appropriately expand the scope of flipped teaching content and explore the appropriate proportion of course content. In the course design, various forms of cross-professional cooperation with clinical doctors should be increased as much as possible, and the contents of flipped classroom should be expanded from basic knowledge to clinical skills.

## Conclusion

Through the application of design thinking in the teaching of histology and embryology courses, we have gained a deeper understanding of its positive impact on innovative teaching strategies, improvement of students' learning experience and academic performance, and the potential value it holds in medical education. We have discovered that the "product" developed through design thinking, namely online flipped teaching, serves as an effective and flexible blended teaching method. It not only enhances students' engagement in learning and fosters their autonomous learning abilities but also encourages both teachers and students to cultivate their innovative capabilities and reshape classroom teaching approaches. Moving forward, further exploration should be undertaken to determine the optimal balance for expanding the content of flipped teaching, to continually uncover its potential in medical education.

## Data Availability

All data generated or analysed during this study are included in this published article.

## Abbreviations

HE: Histology and Embryology
DT: Design Thinking

## References

[1] Cheng X, Ka Ho Lee K, Chang EY, Yang X (2017). The "flipped classroom" approach: Stimulating positive learning attitudes and improving mastery of histology among medical students. Anat Sci Educ.

[2] Cheng X, Chan LK, Li H, Yang X (2020). Histology and Embryology Education in China: The Current Situation and Changes Over the Past 20 Years. Anat Sci Educ.

[3] Magalhaes E, Salgueira AP, Costa P, Costa MJ (2011). Empathy in senior year and first year medical students: a cross-sectional study. BMC Med Educ.

[4] Boyd VA, Whitehead CR, Thille P, Ginsburg S, Brydges R, Kuper A (2018). Competency-based medical education: the discourse of infallibility. Med Educ.

[5] Roberts JP, Fisher TR, Trowbridge MJ, Bent C (2016). A design thinking framework for healthcare management and innovation. Healthc (Amst).

[6] Madson MJ (2021). Making sense of design thinking: A primer for medical teachers. Med Teach.

[7] Scheer A, Noweski C, Meinel C: Transforming Constructivist Learning into Action: Design Thinking in education. Design and Technology Education 2012, 17 3; https://www.researchgate.net/publication/332343908 Transforming Constructivist Learning into Action Design Thinking in education

[8] Gottlieb M, Wagner E, Wagner A, Chan T (2017). Applying Design Thinking Principles to Curricular Development in Medical Education. AEM Educ Train.

[9] IDEO: Design Thinking for Educators Toolkit. https://www.ideo.com/work/toolkit-for-educators.

[10] Badwan B, Bothara R, Latijnhouwers M, Smithies A, Sandars J (2018). The importance of design thinking in medical education. Med Teach.

[11] Deitte LA, Omary RA (2019). The Power of Design Thinking in Medical Education. Acad Radiol.

[12] 2021 EDUCAUSE Horizon Report® _ Teaching and Learning Edition

[13] Chen M, Ye L, Weng Y (2022). Blended teaching of medical ethics during COVID-19: practice and reflection. BMC Med Educ.

[14] Ge L, Chen Y, Yan C, Chen Z, Liu J (2020). Effectiveness of flipped classroom vs traditional lectures in radiology education: A meta-analysis. Medicine (Baltimore).

[15] Awan OA (2021). The Flipped Classroom: How to Do it in Radiology Education. Acad Radiol.

[16] Sertic M, Alshafai L, Guimaraes L, Probyn L, Jaffer N (2020). Flipping the Classroom: An Alternative Approach to Radiology Resident Education. Acad Radiol.

[17] Guo J (2019). The use of an extended flipped classroom model in improving students’ learning in an undergraduate course. J Comput High Educ.

[18] Adams NE (2015). Bloom's taxonomy of cognitive learning objectives. J Med Libr Assoc.

[19] Wagner KC, Byrd GD (2004). Evaluating the effectiveness of clinical medical librarian programs: a systematic review of the literature. J Med Libr Assoc.

[20] Vanka A, Vanka S, Wali O (2020). Flipped classroom in dental education: A scoping review. Eur J Dent Educ.

[21] Eaton M (2017). The flipped classroom. Clin Teach.

[22] Tang F, Chen C, Zhu Y, Zuo C, Zhong Y, Wang N, Zhou L, Zou Y, Liang D (2017). Comparison between flipped classroom and lecture-based classroom in ophthalmology clerkship. Med Educ Online.

[23] Erbil DG (2020). A Review of Flipped Classroom and Cooperative Learning Method Within the Context of Vygotsky Theory. Front Psychol.

[24] Singh K, Mahajan R, Gupta P, Singh T (2018). Flipped Classroom: A Concept for Engaging Medical Students in Learning. Indian Pediatr.

[25] Gomez FC, Trespalacios J, Hsu YC, Yang D (2022). Exploring Teachers' Technology Integration Self-Efficacy through the 2017 ISTE Standards. TechTrends.

[26] Wolcott MD, McLaughlin JE, Hubbard DK, Rider TR, Umstead K (2021). Twelve tips to stimulate creative problem-solving with design thinking. Med Teach.

[27] Bliuc A-M, Goodyear P, Ellis RA (2007). Research focus and methodological choices in studies into students' experiences of blended learning in higher education. The Internet and Higher Education.

[28] de Jong N, van Rosmalen P, Brancaccio MT, Bleijlevens MHC, Verbeek H, Peeters IGP (2022). Flipped Classroom Formats in a Problem-Based Learning Course: Experiences of First-Year Bachelor European Public Health Students. Public Health Rev.

[29] Qureshi SS, Larson AH, Vishnumolakala VR (2022). Factors influencing medical students' approaches to learning in Qatar. BMC Med Educ.

